# Black phosphorus quantum dots prevent atherosclerosis in high-fat diet-fed apolipoprotein E knockout mice

**DOI:** 10.18632/aging.205874

**Published:** 2024-07-10

**Authors:** Yiran Ji, Yilin Wen, Shengwei Zhang, Bingxuan Xu, Shuai Sun, Yun Chen, Xintao Shuai, Tingting Zheng

**Affiliations:** 1 Shenzhen Key Laboratory for Drug Addiction and Medication Safety, Department of Ultrasound, Institute of Ultrasonic Medicine, Peking University Shenzhen Hospital, Shenzhen Peking University-The Hong Kong University of Science and Technology Medical Center, Shenzhen 518036, Guangdong, P.R. China; 2 Department of Cardiology, Peking University Shenzhen Hospital, Shenzhen 518036, Guangdong, P.R. China; 3 PCFM Lab of Ministry of Education, School of Materials Science and Engineering, Sun Yat-Sen University, Guangzhou 510275, Guangdong, P.R. China

**Keywords:** atherosclerosis (AS), black phosphorus quantum dots (BPQDs), nanomedicine, weight loss, autophagy regulation

## Abstract

Atherosclerosis (AS) is the main pathological basis of cardiovascular diseases such as coronary heart disease. Black phosphorus quantum dots (BPQDs) are a novel nanomaterial with good optical properties and biocompatibility, which was applied in the treatment of AS in mice, with good results shown in our previous study. In this study, BPQDs were injected into high-fat diet-fed apolipoprotein E knockout mice as a preventive drug for 12 weeks. Simvastatin, a classic preventive drug for AS, was used as a control to verify the preventive effect of BPQDs. The results showed that after preventive treatment with BPQDs, the plaque area in mice was significantly reduced, the vascular elasticity was increased, and serum lipid levels were significantly lower than those in the model group. To explore the mechanism, macrophages were induced to become foam cells using oxidized low-density lipoprotein. We found that BPQDs treatment could increase cell autophagy, thereby regulating intracellular lipid metabolism. Taken together, these data revealed that BPQDs may serve as a functional drug in preventing the development of AS.

## INTRODUCTION

Among chronic diseases, the leading cause of death worldwide is cardiovascular disease (CVD) [1]. Between 1990 and 2019, mortality and disability rates from CVD continued to rise, resulting in a heavy burden on society and individuals [2]. Atherosclerosis is one of the most common forms of CVD, and its primary factor is endothelial injury. After injury, smooth muscle cells and endothelial cells within the vessel wall upregulate chemokines and adhesion molecules, promoting the mobilization of specific inflammatory cells, such as macrophages and T lymphocytes, into the endothelium. Macrophages internalize oxidized low-density lipoprotein (ox-LDL), which forms foam cells containing large numbers of lipid droplets, which further facilitates LDL retention and the formation of fatty streaks. In response to growth factors and after migrating from the mid-membrane to the intima, smooth muscle cells are not only able to phagocytose lipids and transform into foam cells but can also secrete collagen and other components to form a plaque matrix. Under the above mechanisms, fatty streaks evolve into fibrofatty lesions and fibrous plaques. Plaque formation can easily lead to vascular stenosis, causing inadequate perfusion of vital organs, such as the heart and brain, which can lead to serious CVD adverse events like myocardial infarction and stroke [3–8].

As a chronic disease, CVD remains largely preventable [9]. Two possible scenarios are intervention at an early age and reduction of associated risk factors [1, 10]. Various organizations around the world are also paying increasing attention to the early prevention of CVD. In 2022, the United States Preventative Services Task Force issued a statement on the use of statins for the primary prevention of CVD in adults [11], recommending that statins be used for the primary prevention of CVD. Although statins are widely used in the primary and secondary prevention of CVD, long-term use of statins is often accompanied by statin-related muscle symptoms and neurological symptoms, which lead to poor adherence to statins [12]. A study suggested that for certain populations, the harm of statins outweighs their benefits in primary prevention [13]. Therefore, finding preventive drugs with fewer toxic side effects and better efficacy remains the focus in the prevention and treatment of atherosclerosis.

**Figure 1:**
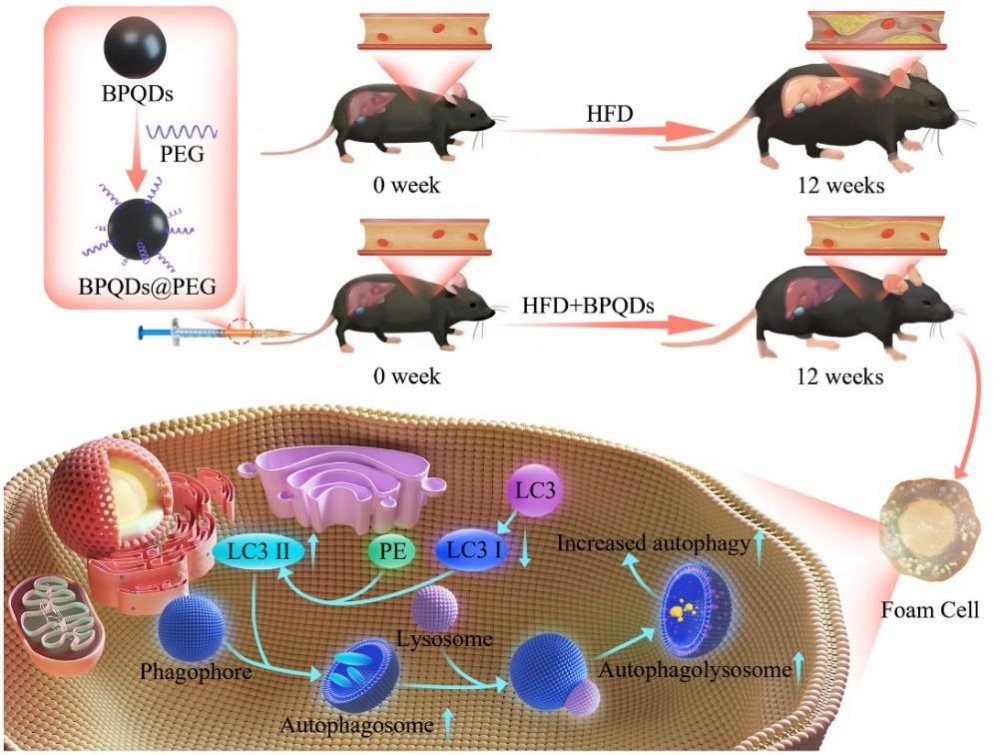
Schematic overview of the development and use of black phosphorus quantum dots (BPQDs) for the prevention of atherosclerosis (AS).

Nanomedicine has become a popular research direction in recent years. Many studies have shown that nanomaterials have many advantages in the treatment of atherosclerosis. Nanoparticles provide numerous possibilities due to their relatively large surface area [14]. For example, nanoparticles can be easily and efficiently captured by monocytes and macrophages without toxicity, thereby improving the bioavailability of the drug. Their retention effect and enhanced permeability can increase the distribution of nanoparticles in atheromatous plaques [15]. As an emerging inorganic nanomaterial, black phosphorus (BP) can be degraded into non-toxic phosphate with excellent biocompatibility and biosafety and has been applied in biomedical fields, such as cancer photothermal/photoacoustic therapy, drug delivery, cancer imaging, and neuronal regeneration [16–19]. As another form of nanomaterials, the ultra-small quantum dot (QD) has quantum confinement and edge effects, which endows it with unique characteristics such as optical effects [20, 21]. In our previous related study, we constructed a mouse atherosclerotic plaque model and injected a 0.1 mL/time (0.1 mg/mL) solution of BPQDs into the tail vein of model mice 3 times/week for 3 consecutive weeks for the first time. After treatment, TC and LDL of serum decreased and HDL increased (Supplementary Figure 1). Additionally, we discovered a considerable reduction in the plaque area of the aorta in the atherosclerotic mouse model after BPQDs therapy (Supplementary Figure 2), which proved that BPQDs had a certain therapeutic effect on atherosclerosis with little toxic or side effects. Therefore, based on our previous results, we investigated the use of BPQDs as preventive drugs for atherosclerosis and explored the preventive effect of BPQDs on atherosclerosis (Figure 1).

## RESULTS

### Characterization of BPQDs

**Figure 2:**
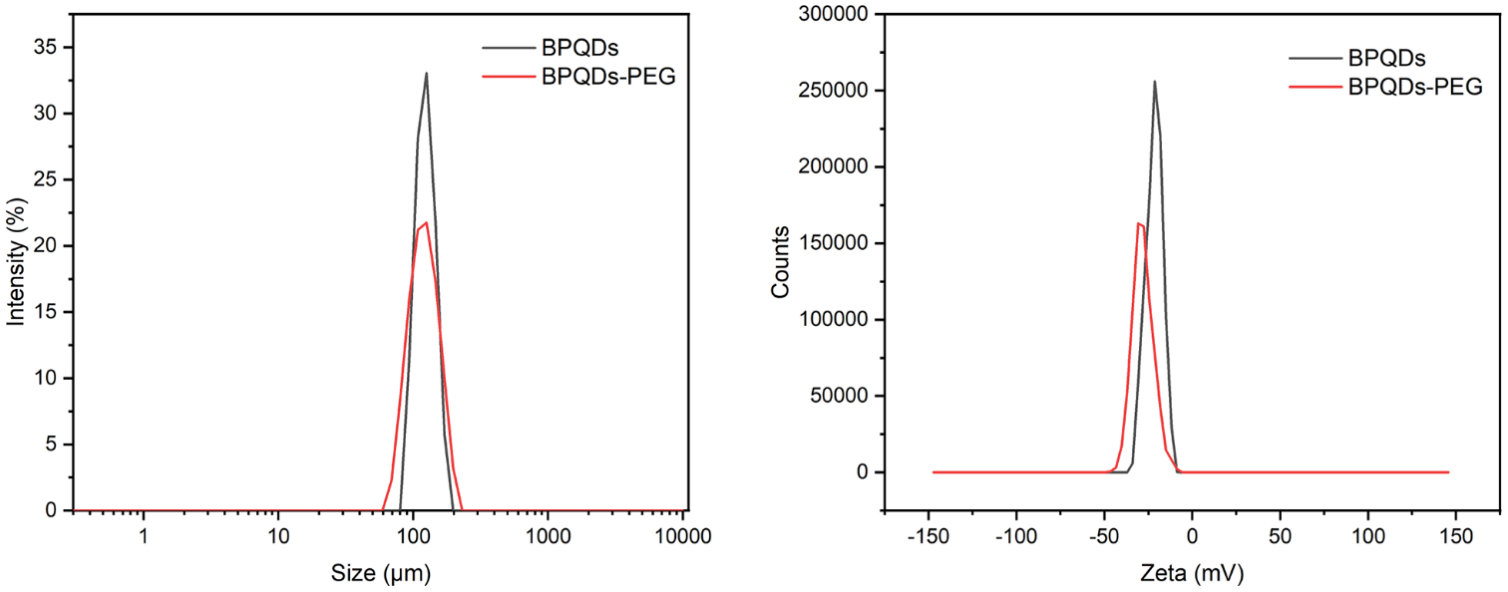
Particle size distribution and zeta potential of black phosphorus quantum dots.

First, we used dynamic light scattering (DLS) to perform particle size characterization of BPQDs. DLS showed that the BPQDs had good dispersibility, with a particle size of 121.8 nm and a zeta potential of −21.74 mV. After PEG modification, the particle size was 130.5 nm and the zeta potential was −28.48 mV (Figure 2). Using PEG coating on the surface of nanoparticles is a common method to improve drug delivery, and PEGylation of nanoparticles can prolong systemic circulation time and improve the curative effect [22, 23]. Our results showed that the particle size and absolute potential of the BPQDs increased slightly after PEGylation, suggesting that the structure is more stable after PEGylation as the zeta potential can affect how much the nanoparticles penetrate the membrane.

### Preventive effects of BPQDs *in vivo*

**Figure 3:**
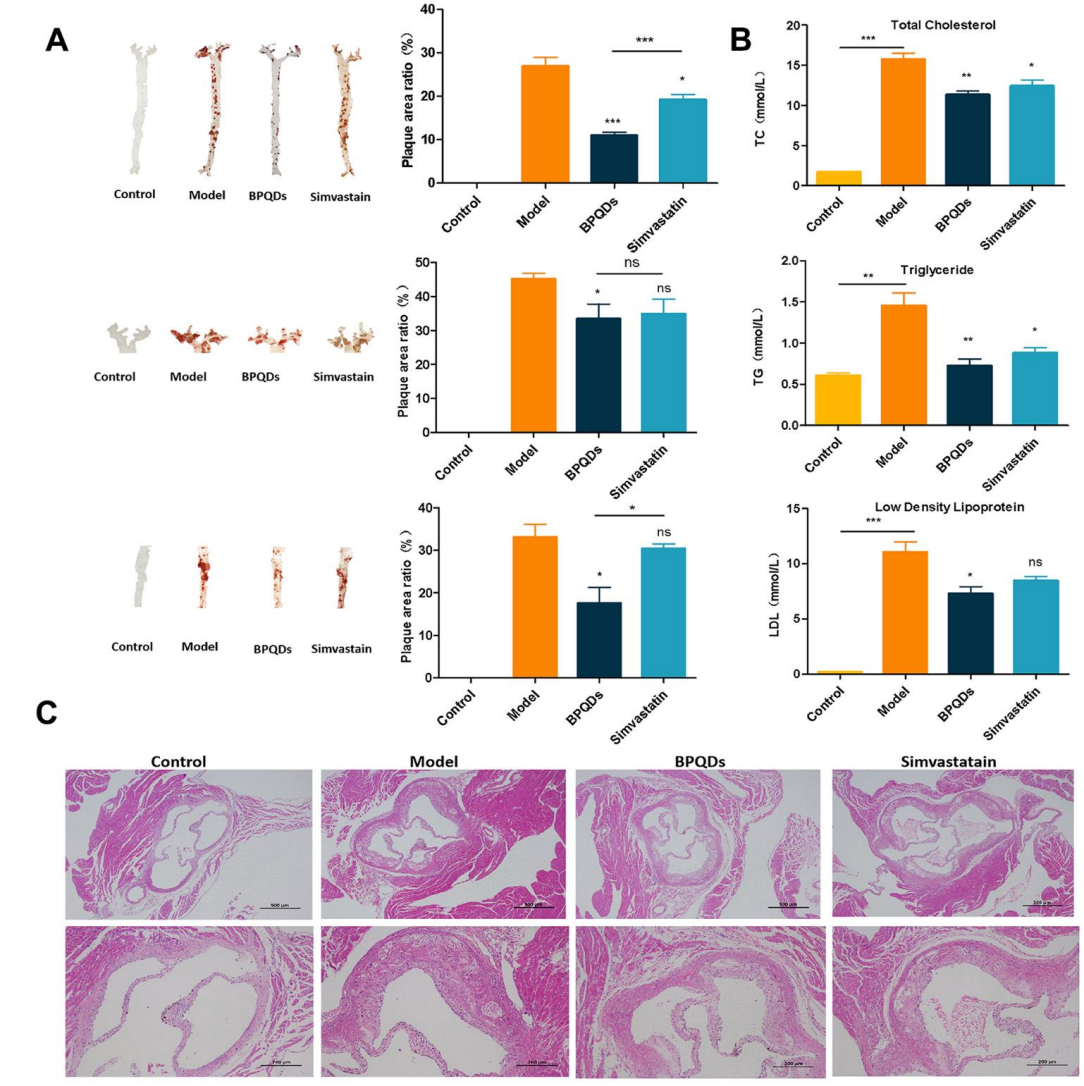
**Preventive effect of each group after 12 weeks of preventive drug use.** (**A**) Oil red O staining of aortic tree, aortic arch, and abdominal aorta, and the proportion of plaque area; statistical analysis results were obtained by comparison with the model group (**B**) Comparison of three levels of blood lipids in each group; statistical analysis results were obtained by comparison with the model group (**C**) HE staining of aortic root in each group (first row: 40×, second row: 100×).

After 12 weeks of consuming a high-fat diet, the model group had significantly more plaque area than the control group that consumed a normal diet. After 12 weeks of drug prophylaxis, the plaque area in the BPQDs prophylaxis group and simvastatin prophylaxis group was smaller than that in the model group, and the difference was statistically significant. Among them, there was less aortic plaque formation in the BPQDs prophylaxis group than in the simvastatin prophylaxis group; there was also a significant reduction in the plaque area in the BPQDs prophylaxis group within the aortic arch and abdominal aorta (Figure 3A). This indicates that both statins and BPQDs have a certain preventive effect against atherosclerosis, and the preventive effect of BPQDs against plaque formation is better than that of simvastatin.

In the wholly and locally enlarged view of pathological sections of the aortic root, the aortic wall of mice in the model group was significantly thickened, with more lipid infiltration, whereas the aortic wall of mice in the BPQDs and simvastatin groups was thinner with less lipid infiltration (Figure 3C). The three biochemical indexes of blood lipids indicated that the TC, TG, and LDL in the BPQDs and simvastatin prophylaxis groups were significantly reduced compared with those in the model group (Figure 3B). This further indicated that the BPQDs treatment group had a reduced accumulation of lipids.

**Figure 4:**
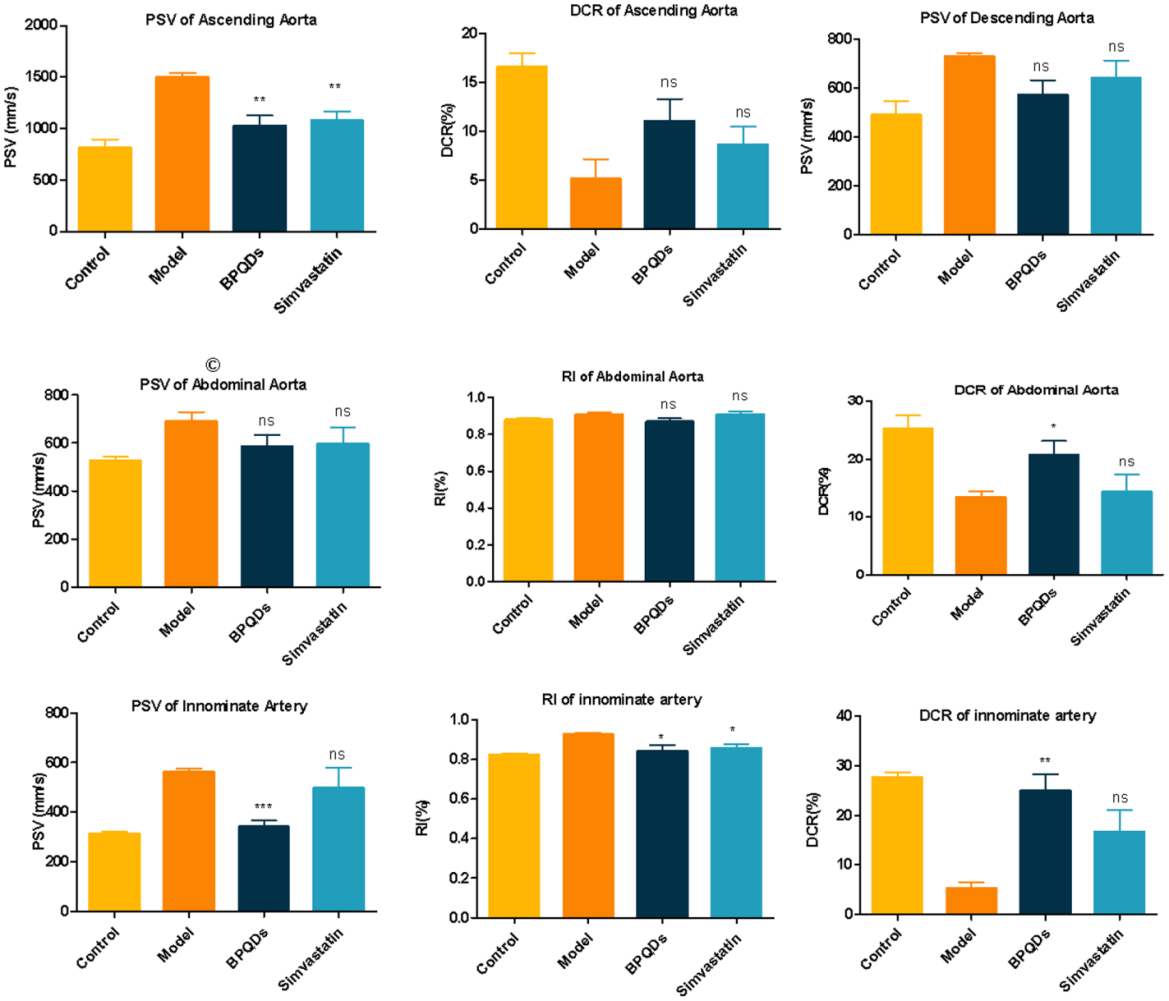
**Comparison of blood flow parameters of ascending aorta, descending aorta, abdominal aorta, and innominate artery in each group after 12 weeks of preventive medication.** Statistical analysis results were obtained by comparison with the model group.

Ultrasound was used to detect arterial and hemodynamic changes in mice of different groups. We found that changes in the ascending aorta PSV, ascending aorta DCR, abdominal aorta DCR, innominate artery RI, innominate artery DCR, and innominate artery PSV in the BPQDs prophylaxis group were statistically significant compared with those in the model group. In the simvastatin group, only the reduction of PSV in the ascending aorta was statistically significant compared with the model group (Figure 4). Ultrasound results suggest that BPQDs may improve the elasticity of atherosclerotic blood vessels and reduce blood flow resistance. The effect of BPQDs on Improving hemodynamics was better than that of simvastatin.

### Safety evaluation of BPQDs

**Figure 5:**
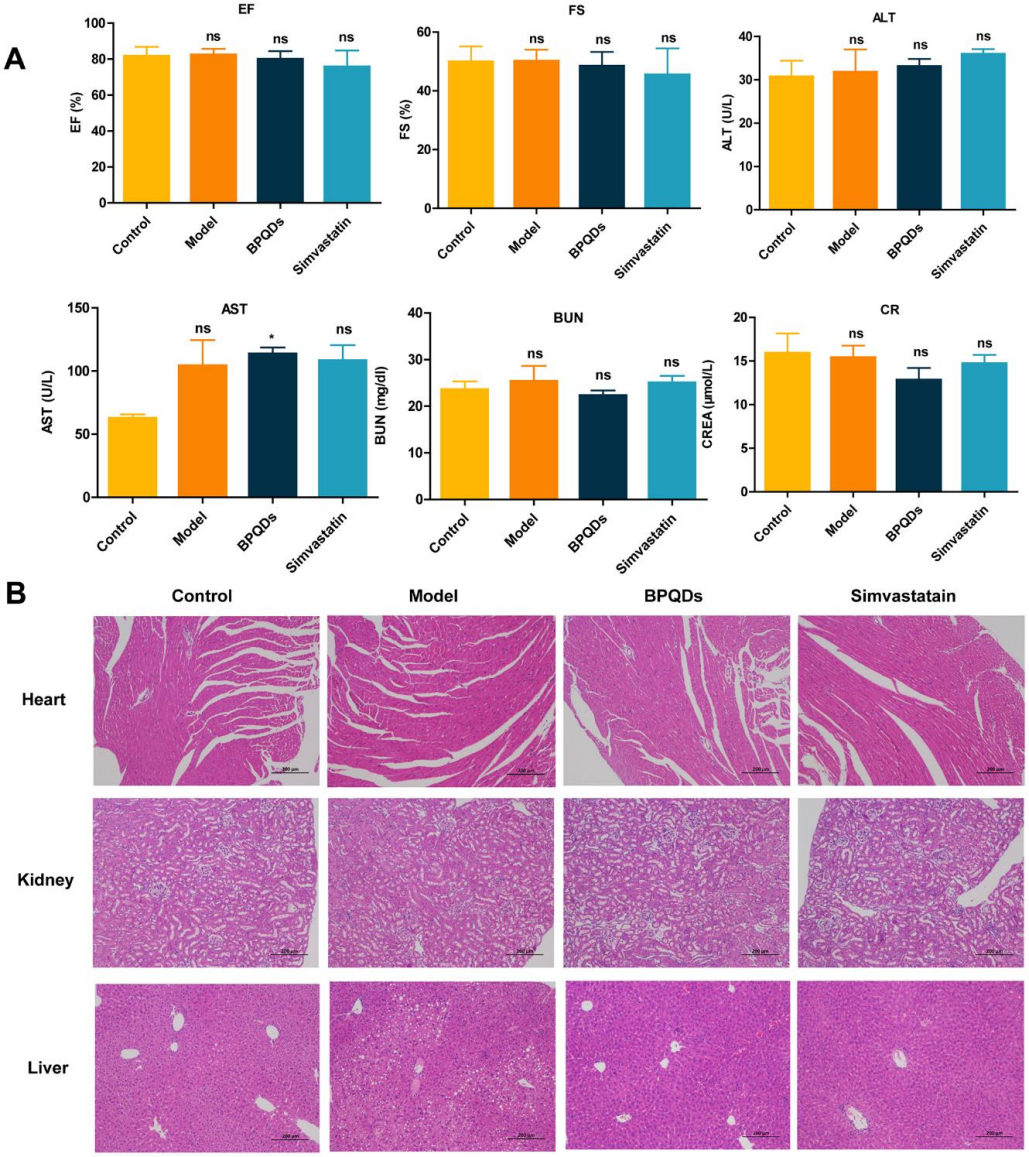
**Safety evaluation of the drug after 12 weeks of prophylactic administration.** (**A**) Comparison of cardiac and liver and kidney function in each group. (**B**) HE staining of the heart, liver, and kidney in each group (100×).

In terms of safety *in vivo*, cardiac function in the BPQDs and simvastatin prophylaxis groups was within the normal range, and blood biochemical indexes, including transaminase and urea nitrogen, showed that the liver and kidney function was within the normal range (Figure 5A). Pathological HE staining showed no abnormal manifestations, such as structural damage in the cardiomyocytes, liver lobular structure, and sections of kidney tissue (Figure 5B), with zero deaths occurring while taking prophylactics, indicating that the administration of BPQDs and simvastatin for 3 consecutive months is safe. In addition, the results of liver HE staining showed that the BPQDs and simvastatin groups had less hepatic adipose vacuole infiltration than the model group, and both drugs could alleviate hepatic steatosis.

### Exploration of the mechanism of BPQDs in preventing atherosclerosis

**Figure 6:**
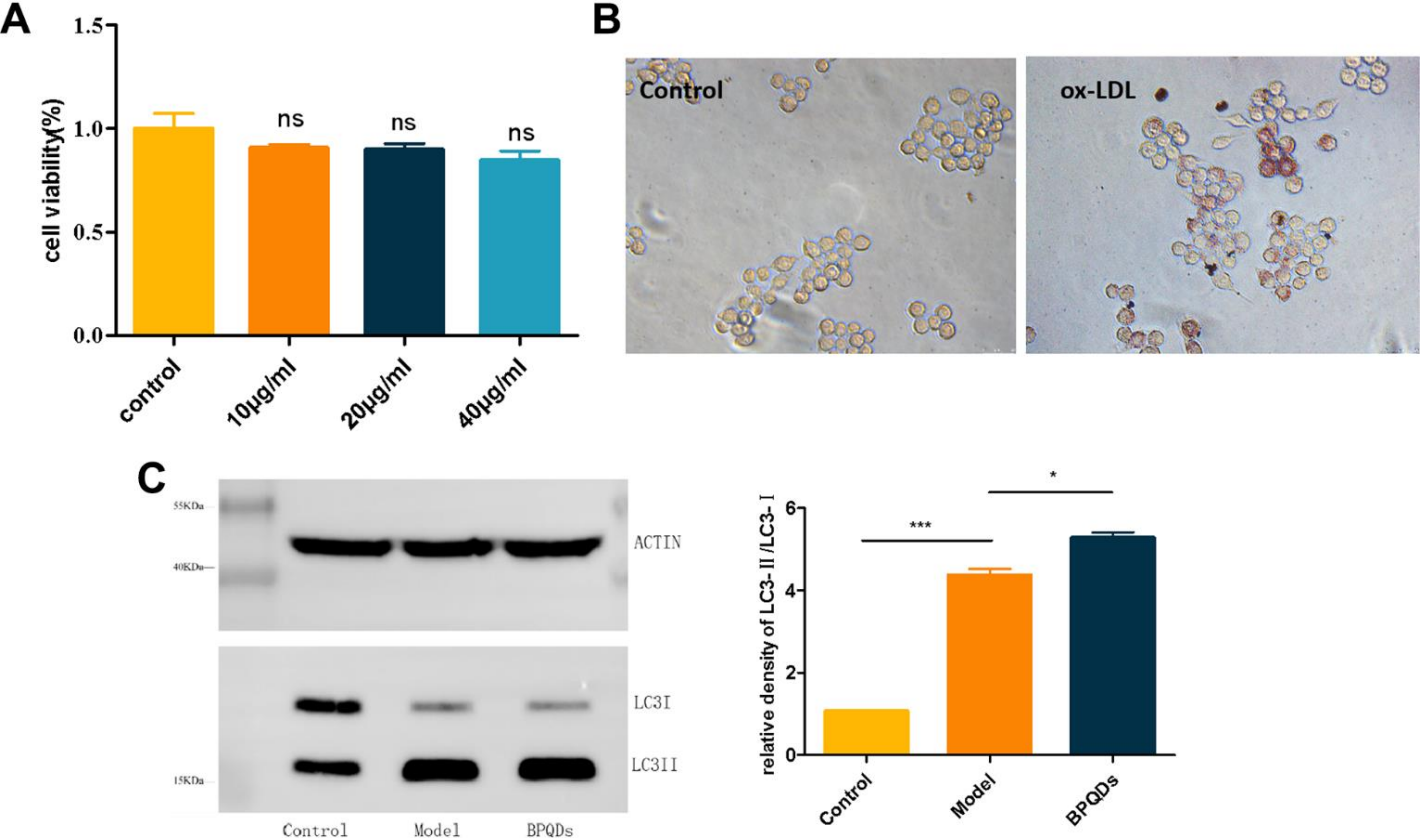
**Results of the mechanism of black phosphorus quantum dots in preventing atherosclerosis.** (**A**) *In vitro* safety assessment of CCK8. (**B**) Oil red O staining of RAW264.7 cells after ox-LDL induced foam cells (20). (**C**) Expression of autophagy protein LC3 detected by western blot.

First, we assessed the toxicity of BPQDs *in vitro*. The results of CCK8 assay showed that the cell survival rate of 10 μg/mL, 20 μg/mL, and 40 μg/mL BPQDs was not statistically significant compared with that of the control group, and no toxic reaction was induced in cells (Figure 6A). RAW264.7 cells were induced into a foam cell model using 100 μg/mL ox-LDL, and oil red O staining verified that the foam cells were successfully modeled and lipids successfully entered the cells (Figure 6B). Then, a solution of 20 μg/mL BPQDs was used to evaluate the expression of autophagy-associated protein LC3. As shown in Figure 6C, the β-actin in all groups was basically consistent. The LC3II/LC3I ratio was increased in the model group after ox-LDL induction (Figure 6C), and the results were statistically significant. Autophagy is a self-protection mechanism of cells. LC3II expression in the 20 μg/mL BPQDs prophylaxis group was higher than that in the model group. LC3I expression was lower than that in the model group, the LC3II/LC3I protein ratio was increased, and the difference was statistically significant, suggesting that BPQDs can promote autophagy to regulate lipid levels.

### Weight reduction effect of BPQDs

**Figure 7:**
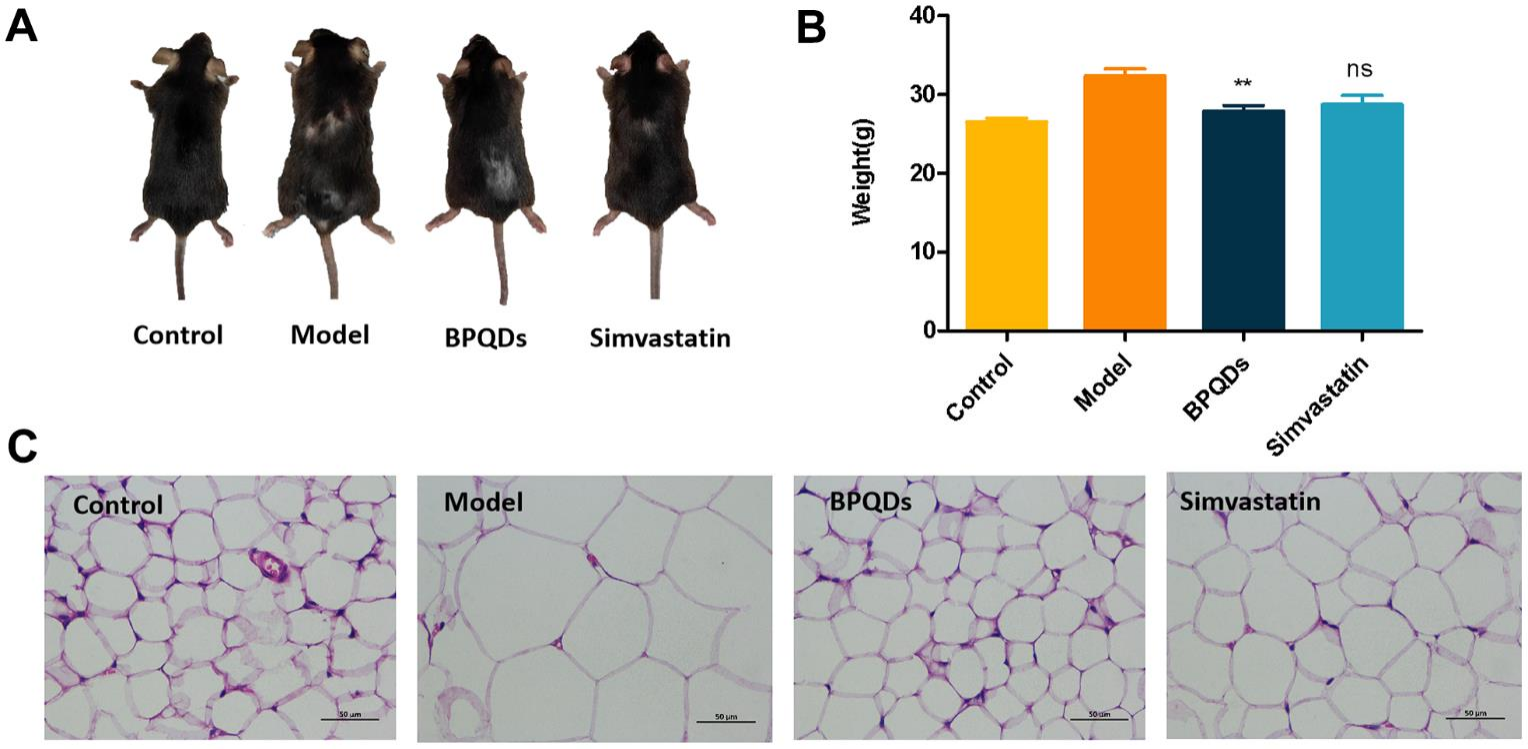
**Effect of black phosphorus quantum dots on weight loss after 12 weeks of preventive administration.** (**A**) Comparison of body size of mice in each group. (**B**) Comparison of body weight of mice in each group; statistical analysis results were obtained by comparison with the model group. (**C**) HE staining of visceral cells of mice in each group (400×).

After 12 weeks of administration, we analyzed the weight of mice in each group. Mice in the model group were the heaviest; mice in the BPQDs prophylaxis group showed statistically significant weight loss, compared with the model group. Mice on a high-fat diet with simvastatin administration did not lose weight significantly (Figure 7B). HE staining of visceral fat cells showed that the volume of visceral fat cells among mice in the BPQDs prophylaxis group was significantly smaller than that in the model group, which was close to that of the control group. The volume of visceral fat cells in mice on a high-fat diet administered simvastatin was smaller than that of the model group, and that of the BPQD prophylaxis group was smaller than those of the other two groups (Figure 7C). Thus, BPQDs can cause weight loss by reducing the volume of visceral fat cells. This is the first time that BPQDs have been found to be universally effective in reducing fat and weight.

## DISCUSSION

Atherosclerosis is a common form of CVD, the main components of which are lipid accumulation in the large arteries and inflammatory responses [3]. Simply improving treatment methods cannot reduce the global burden of CVD [24]. As a type of chronic disease, early intervention is an important measure to prevent and treat atherosclerosis and reduce the social burden [25]. The primary factors in atherosclerosis are vascular endothelial injury, release of inflammatory factors, and abnormal lipid accumulation in atherosclerotic lesions, leading to plaque formation [4]. Lipid metabolism disorder is a key factor in the development of atherosclerosis [26], especially the accumulation of LDL in the arterial wall, which is considered a pathogenic factor in atherosclerosis [27]. Both TC and LDL are thought to be strongly associated with the development of atherosclerosis [28]. Although not as strongly associated with CVD as cholesterol and LDL, TGs are considered an independent risk factor for CVD as well [29]. Therefore, reducing lipid accumulation is a necessary measure to prevent atherosclerosis.

Statins competitively inhibit 3-hydroxy-3-methyl glutaryl coenzyme A reductase (HMG-CoA reductase), which reduces endogenous cholesterol synthesis. The ACC/AHA, the European Society of Cardiology, and the Canadian Cardiovascular Society all recommend statins for the prevention of atherosclerotic heart disease in people at high risk [30–32]. Therefore, simvastatin, which is commonly used clinically, was selected as a positive control to verify the preventive effect of BPQDs. Serologically, both simvastatin and BPQDs reduced cholesterol and TGs in atherosclerotic mice; however, BPQDs reduced serum LDL more effectively than simvastatin. In general, the plaque area percentage in model mice after BPQDs prophylaxis was smaller than that after simvastatin prophylaxis. Changes in local hemodynamic characteristics can also lead to the development of atherosclerosis. Studies have shown that carotid EDV, PSV, and RI are positively correlated with the development of CVD [33, 34]. The higher these indicators, the higher the incidence of CVD. Our results also showed that BPQDs have superior effectiveness to simvastatin in terms of hemodynamic changes. The results showed that the preventive effect of BPQDs was better than that of simvastatin.

Because the preventive effect of BPQDs was better than that of statins in various indexes, we explored the mechanism of BPQDs in the prevention and treatment of atherosclerosis. The development of atherosclerosis is closely related to foamy macrophages; therefore, in exploring the mechanism of atherosclerosis prevention and treatment, we mainly focused on the autophagy of macrophages. Autophagy is a cellular pathway that depends on lysosomes, a process that degrades abnormal cellular substances such as damaged organelles and misfolded proteins. There are three main forms of autophagy: chaperone-mediated autophagy, macroautophagy, and microautophagy. Among them, macroautophagy is the most common form of autophagy. In macroautophagy, phagocytic vesicles phagocytose specific abnormal substances and form autophagosomes, then these autophagosomes are transported to intracellular lysosomes; this is the main metabolic pathway used by eukaryotes to degrade long-lived proteins and organelles. Autophagy is not only a protective mechanism but is also a process of “garbage sorting” [35–38]. Studies have shown a positive correlation between inhibition of autophagy and lipid accumulation in atherosclerosis [39]. At the cellular level, macrophages phagocytose lipids and become foam cells, which leads to lipid accumulation. The lipids encapsulated in foam cells are mainly free cholesterol and cholesterol esters, which are stored in the form of lipid droplets. Autophagosomes can encapsulate lipid droplets, which are then transported to lysosomes for degradation, promoting lipid outflow from foam cells, thereby reducing lipid accumulation and slowing the progression of atherosclerosis [40]. As a chronic inflammatory disease, chronic stress in the pathophysiological process of atherosclerosis may lead to autophagy dysfunction; thus, one anti-inflammatory approach is to improve the level of autophagy. Various receptors in autophagy, such as Nod-like receptors and Toll-like receptors, identify characteristic inflammatory factors and bind them to the autophagosome to inhibit the production of inflammasomes and to coordinate the removal of exogenous and endogenous sources of inflammation [41, 42]. In summary, autophagy alleviates the development of atherosclerosis by promoting intracellular lipoprotein degradation and cholesterol transport and reducing inflammation. Activation of the autophagy pathway may be a potential therapeutic strategy to prevent formation of the necrotic core in atherosclerotic lesions [43].

Autophagy is co-regulated by a variety of genes and proteins, the most important of which are autophagy-associated genes (ATG). More than 30 proteins encoded by ATG participate in the whole process of autophagy [44]. The microtubule-associated protein light chain 3 (LC3) is encoded by the ATG8 [45] and is often used as a marker of autophagy. On the one hand, ATG12 covalently binds with ATG5 under the action of ATG7 and ATG10 to form the conjugate of ATG12-ATG5, which promotes the conjugation of LC3 I with phosphatidylethanol-amine (PE). On the other hand, with the assistance of ATG4, ATG7, and ATG3, LC3 is cleaved into LC3 I, which covalently binds to PE on the membrane to form lipidized LC3 II [46]. During autophagy, LC3 I is transformed into the membrane-bound form LC3 II, which promotes autophagy; the LC3 II/I ratio can be used to estimate the level of autophagy [39, 47, 48]. Macrophages were incubated with ox-LDL to construct a foam cell model, which has been used in many studies [49, 50]. Western blot results showed that the LC3 II/I ratio of the BPQDs group was increased. Our study suggests that BPQDs can prevent the progression of atherosclerotic disease by promoting autophagy to regulate lipid levels and reduce inflammatory responses.

In the process of prophylactic administration, we also found that BPQDs can reduce the volume of visceral fat and have a weight loss effect, which may be related to their role in regulating lipids. This discovery may be used in the development of obesity treatment as well as weight loss and fitness supplements.

## MATERIALS AND METHODS

### Materials

BPQDs were purchased from XFNANO (Nanjing, Jiangsu, China). RAW264.7 cells were obtained from the Laboratory Center of Shenzhen University. Cell medium high glucose DMEM basic and fetal bovine serum were purchased from Gibco (Carlsbad, CA, USA). Anti-LC3 antibody was from Sigma (St. Louis, MO, USA).

### Preparation of BPQDs

We took an appropriate amount of 0.5 mg/mL BPQDs solution for ultrasonic disruption, centrifuged the solution (12,500 rpm, 18 min) to remove the solvent, and then added PEG solution with a corresponding volume of 1 mg/mL (the PEG solution was prepared with ddH2O). The corresponding volume calculation was based on the principle of 1:2 mass ratio of BPQDs to PEG. Centrifugation was performed again (12,500 rpm, 18 min), and the corresponding volume of normal saline was added to prepare the solution with a concentration of 0.1 mg/mL BPQDs. Finally, the solution was mixed with ultrasonic disruption for 3–5 min, resulting in a light brown solution. The BPQDs solution was mixed and used immediately. The entire process took place away from light, and the solution was mixed repeatedly during use. BPQDs solution at the remaining concentrations was obtained using a step-down concentration method.

### Cell culture

RAW264.7 cells were grown in complete medium containing 10% fetal bovine serum, 100 U/mL penicillin, and 100 mg/mL streptomycin with high-glucose DMEM. The cells were cultured in an incubator at 37° C and 5% CO_2_ with a passage ratio of 1:3.

### Detection of drug toxicity by the CCK8 method

The experimental group was randomly divided into five groups: the blank group (no cells), control group (equal volume PBS solution), 10 μg/mL BPQDs solution group, 20 μg/mL BPQDs solution group, and 40 μg/mL BPQDs solution group. We cultured the cells for 24 h after administration and added CCK8 solution. The absorbance at 450 nm was measured using a microplate reader. Cell viability was calculated when the control optical density (OD) was close to 1 according to the following formula: cell survival rate = (OD experimental group − OD blank)/(OD control group − OD blank group) × 100%.

### Western blot

We lysed RAW264.7 cells with RIPA (Beyotime, Shanghai, China) containing 1 mmol/L phenylmethylsulfonyl fluoride (PMSF), and then, the cells were centrifuged and boiled at 99° C for 10 min to obtain total proteins. The proteins were analyzed on SDS-PAGE and transferred to a polyvinylidene fluoride membrane. After being sealed with 5% milk at room temperature for 1 h, proteins were combined with anti-rabbit LC3 antibody (1:1000) at 4° C overnight, and β-actin antibody was used as a control. After secondary antibody incubation, the membranes were washed three times with TBST. Amersham Image Quant 800 was used for the visualization of immunoblots.

### Animal experiment

### Animal preparation

Healthy, male C57BL/6J mice (n=6) and ApoE^-/-^ mice (n=18) (all 5–6 weeks old) were purchased from Guangdong Yaokang Biological Technology Co., Ltd, China. All experiments were approved by the Ethics Committee of Peking University Medical Center, Shenzhen, and all experiments were conducted at Peking University Medical Center, Shenzhen. C57BL/6J mice formed a control group (n=6). ApoE^-/-^ mice were randomly divided into a model group (n=6), a BPQDs prophylaxis group (n=6), and a simvastatin prophylaxis group (n=6). The control group was fed a normal diet (ordinary feed). ApoE^-/-^ mice were fed a high-fat diet (40 kcal% fat and 1.25% cholesterol) with water freely available. On the date when the high-fat diet was given, both the control group and the model group were administered 0.1 mL/time of 0.9% normal saline injected via the tail vein. The BPQDs prophylaxis group was administered 0.1 mL/time of BPQDs solution (concentration 0.1 mg/mL). The simvastatin prophylaxis group was administered 0.1 mL/time of simvastatin solution (concentration 0.1 mg/mL). Both were injected intravenously via the tail vein of the mice, three times a week on alternate days for 12 weeks. Relevant data of all experimental mice were collected at the end of 12 weeks’ prophylactic administration.

### Ultrasonic detection of major artery indexes and cardiac function

One day after the last administration on week 12, we used the MS400 probe of a Vevo2100 small animal ultrasound instrument (frequency 18 Hz) to detect and measure the indicators below: diastolic diameter; systolic diameter; peak systolic flow rate (PSV); peak diastolic flow rate (EDV) of the abdominal aorta; diastolic diameter, systolic diameter, and PSV of the ascending aorta; diastolic diameter, systolic diameter, PSV, and EDV of the innominate artery; PSV of the descending aorta; and left ventricular end-diastolic diameter (LVEDD) and left ventricular end-systolic diameter (LVESD). We calculated the vascular diameter change rates (DCR = [minimum systolic diameter − maximum diastolic diameter] × 100/minimum systolic diameter) and resistance index (RI = (PSV− EDV)/PSV) of the ascending aorta, abdominal aorta, and innominate artery. The left ventricular short axis shortening rate (FS) was calculated as follows:


$$\begin{array}{l} \text{LVEDV}=\frac{7\;\times \;{\text{LVEDD}}^{3}}{2.4+\text{LVEDD}}\\ \text{LVESV}=\frac{7\;\times \;{\text{LVEDSD}}^{3}}{2.4+\text{LVESD}} \end{array}$$

$$\text{FS}=\frac{\text{LVEDD}-\text{LVESD}}{\text{LVEDD}}\times 100\%$$

$$\text{EF}=\frac{\text{LVEDV}-\text{LVESV}}{\text{LVEDV}}\times 100\%$$

### Biochemical indicators

On the day before the end of the experiment, the mice were freely given drinking water and fasted for at least 12 h. The mice were anesthetized via intraperitoneal injection of 1% pentobarbital sodium according to body weight (70 mg/kg). Blood was taken from the inferior vena cava. After centrifugation (3000 rpm) for 10 min, the serum was collected, placed in a new Eppendorf tube, and stored at −80° C. The labeled serum samples were sent to Wuhan Servicebio Biological Technology Co., Ltd. for testing serum lipid levels (total cholesterol [TC], triglyceride [TG], LDL-C, and high-density lipoprotein cholesterol [HDL-C]), and liver and kidney functions (AST, ALT, BUN, and CR) were assessed.

### Tissue sections and aortic tree plaque staining

The mice were sacrificed at the end of the experiment, and then, heart lavage was performed to clean the blood vessels as much as possible. The visceral adipose tissue, liver, kidney (stripped renal envelope), and heart were removed and stored in formalin and sent to Wuhan Servicebio Biological Technology Co., Ltd for testing. After the organs were removed, we carefully separated the whole aorta. The three branches of the aortic arch, the thoracoabdominal aorta, and the bilateral common iliac artery were exposed as much as possible. After dissection, the aorta was placed under an Olympus SZX10 stereo microscope, the fatty tissue and connective tissue on the surface were stripped away, and then, the aorta was stained with oil red O. We made sure that all colored plaques were inside the arteries and then opened the aorta longitudinally and laid them flat on a slide for photographing. The photographs were spliced with Adobe Photoshop; after splicing, the photographs were analyzed with Image J to determine the proportion of plaque in the entire artery. Then, the aortic arch and abdominal aorta were dissected out, and the proportions of plaque area in the aortic arch and abdominal aorta were calculated in the same way.

### Statistical analysis

IBM SPSS 26.0 and GraphPad Prism 5 were used for statistical analysis. Excel, ImageJ, Origin, and Photoshop were used to draw the images. The sample data obtained in the experiment were averaged three times and expressed as mean ± standard deviation. Comparisons between two groups were made using a t-test or one-way analysis of variance. Differences with *P*<0.05 (two-sided) were considered statistically significant.

## Supplementary Material

Supplementary Figures
