# Supplementary material for: Black phosphorus quantum dots prevent atherosclerosis in high-fat diet-fed apolipoprotein E knockout mice
Source: Aging (Albany NY). 2024 Jul 10;16(13):10784–98. doi: 10.18632/aging.205874 (PMC11272127; doi:10.18632/aging.205874)
Supplement: Supplementary Figures [file aging-16-205874-s001.pdf]

SUPPLEMENTARY FIGURES

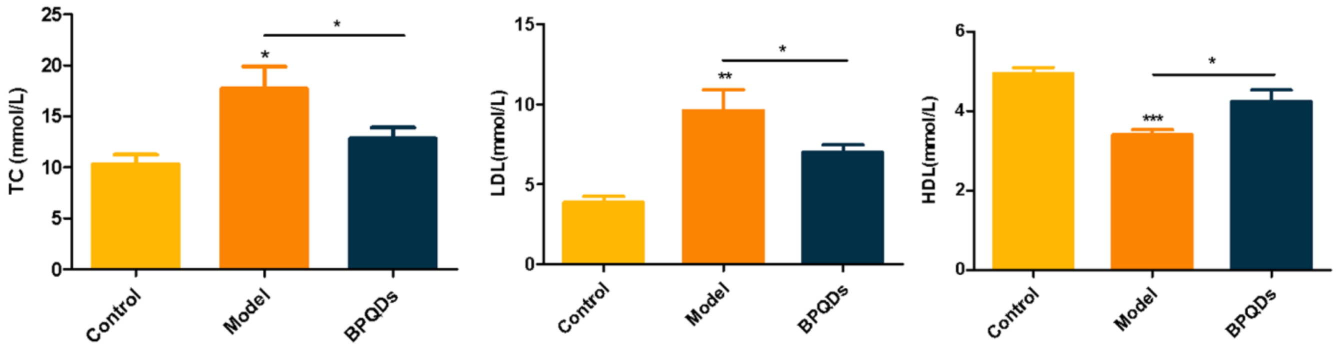

Supplementary Figure 1. Serum blood lipids index of mice.

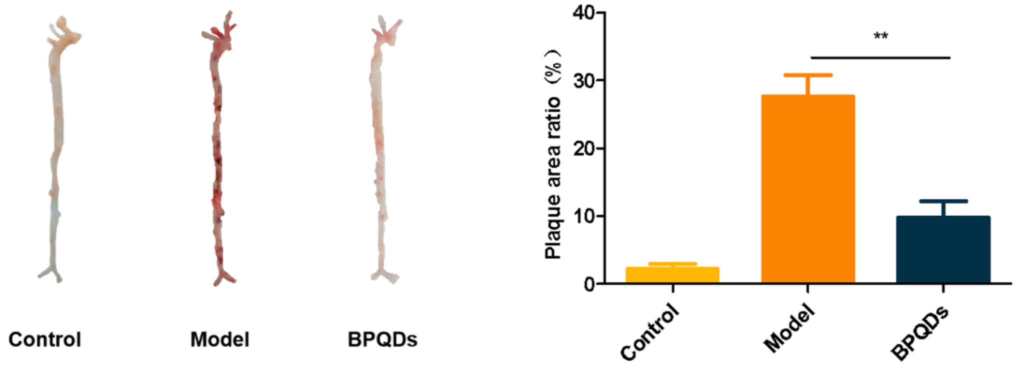

Supplementary Figure 2. Aorta oil red O staining of aorta and corresponding statistical analysis.
